# In vivo flow cytometry reveals a circadian rhythm of circulating tumor cells

**DOI:** 10.1038/s41377-021-00542-5

**Published:** 2021-05-28

**Authors:** Xi Zhu, Yuanzhen Suo, Yuting Fu, Fuli Zhang, Nan Ding, Kai Pang, Chengying Xie, Xiaofu Weng, Meilu Tian, Hao He, Xunbin Wei

**Affiliations:** 1 State Key Laboratory of Oncogenes and Related Genes, Shanghai Cancer Institute, Med-X Research Institute and School of Biomedical Engineering, Shanghai Jiao Tong University, 200030 Shanghai, China; 2 Biomedical Pioneering Innovation Center, Peking University, 100871 Beijing, China; 3 School of Life Sciences, Peking University, 100871 Beijing, China; 4 School of Instrument Science and Optoelectronics Engineering, Beijing Information Science and Technology University, 100192 Beijing, China; 5 Biomedical Engineering Department, Peking University, 100081 Beijing, China; 6 Key Laboratory of Carcinogenesis and Translational Research (Ministry of Education/Beijing), Peking University Cancer Hospital and Institute, 100142 Beijing, China

**Keywords:** Biophotonics, Optical techniques

## Abstract

Circulating tumor cells (CTCs) is an established biomarker of cancer metastasis. The circulation dynamics of CTCs are important for understanding the mechanisms underlying tumor cell dissemination. Although studies have revealed that the circadian rhythm may disrupt the growth of tumors, it is generally unclear whether the circadian rhythm controls the release of CTCs. In clinical examinations, the current in vitro methods for detecting CTCs in blood samples are based on a fundamental assumption that CTC counts in the peripheral blood do not change significantly over time, which is being challenged by recent studies. Since it is not practical to draw blood from patients repeatedly, a feasible strategy to investigate the circadian rhythm of CTCs is to monitor them by in vivo detection methods. Fluorescence in vivo flow cytometry (IVFC) is a powerful optical technique that is able to detect fluorescent circulating cells directly in living animals in a noninvasive manner over a long period of time. In this study, we applied fluorescence IVFC to monitor CTCs noninvasively in an orthotopic mouse model of human prostate cancer. We observed that CTCs exhibited stochastic bursts over cancer progression. The probability of the bursting activity was higher at early stages than at late stages. We longitudinally monitored CTCs over a 24-h period, and our results revealed striking daily oscillations in CTC counts that peaked at the onset of the night (active phase for rodents), suggesting that the release of CTCs might be regulated by the circadian rhythm.

## Introduction

Circulating tumor cells (CTCs) have aroused wide attention as indicators of metastasis^1–3^. CTCs are very rare cells that are shed by solid tumors and circulate in the vasculature. A small number of them can settle and colonize a new site to form metastasis. Analyses of CTCs have provided insights into the mechanism of metastasis, thereby facilitating the development of early diagnosis, prognosis evaluation, and antimetastatic therapeutic strategies^4,5^. The temporal distribution and circulation dynamics of CTCs are important for understanding the mechanisms underlying tumor cell dissemination. Although studies have revealed that the circadian rhythm may disrupt the growth of tumors^6–8^, it is generally unclear whether the circadian rhythm controls the trafficking of CTCs. The circadian rhythm is the internal molecular clock that oscillates with a periodicity of 24 h under the entrainment of external or environmental cues. These cues are known as zeitgebers, meaning environmental agents or events that provide the stimulus for setting or resetting the biological clock of an organism. Light is a major environmental zeitgeber that entrains the biological clock of the suprachiasmatic nucleus^9^. Given that circadian rhythm regulates multiple pathways related to cancer development, such as metabolism, hormone secretion, and cell division^10,11^, it is possible that CTC release may be controlled by the circadian clock.

CTCs are not easy to detect because they are rare in the peripheral blood, at an estimated concentration of 1–10 CTCs per mL^12^. It is difficult to monitor CTCs over a long period of time. For most of the current detection methods in biomedical research and clinical examinations, it is necessary to draw blood first^13,14^. The fundamental principle of these blood-based in vitro detection methods is that blood samples are representative; which assumes that the CTC count does not change significantly over the course of several days. However, the distribution of CTCs in circulation may not be uniform^15,16^. Patients with zero CTCs detected at a given time point may not be CTC-free^17^. However, it is not practical to draw blood from patients repeatedly to clarify the temporal distribution of CTCs. A feasible strategy for investigating this issue is to monitor CTCs in animal models via in vivo detection methods.

Fluorescence in vivo flow cytometry (IVFC) is an optical technique that is able to detect moving cells directly in living animal models in a noninvasive and continuous way^18–20^. By detecting fluorescence from a light sheet across an artery in the experimental animal (details in “Materials and Methods” section), it allows serial measurements of fluorescently labeled CTCs over minutes to hours and provides temporal information. Compared with in vitro detection methods of CTCs, blood drawing is avoided with fluorescence IVFC. Additional processes involved in vitro detection methods, such as cell lysis, centrifugation, and enrichment, are avoided as well. These in vitro processes could perturb the biological environments of CTCs and significantly affect the accuracy of CTC detection. Fluorescence IVFC was first developed by Lin et al. at the Massachusetts General Hospital in Boston. It has been widely applied for detecting the CTCs of leukemia^21^, liver cancer^22^, prostate cancer^23^, breast cancer^24^, etc^25^. It has unique advantages in the investigation of the circadian rhythm of CTCs, and this application has not been reported previously.

In this study, we utilized fluorescence IVFC to explore the temporal distribution and circulation dynamics of CTCs by analyzing the changes in CTC counts over the course of minutes to hours. We established an orthotopic mouse model of human prostate cancer and monitored CTCs over 24 h. It was found that CTCs were not uniformly distributed and tended to occur in stochastic bursts at the early stages of cancer. Prostate cancer CTCs fluctuated markedly over 24 h, peaking at the onset of night. Moreover, we also provided sampling guidelines to improve detection accuracy by analyzing the circadian distribution profile and short-term variation in CTCs. Our results suggest that the trafficking of CTCs may be regulated by circadian rhythm and thus provide new insights into the mechanism of CTC release.

## Results

### CTCs monitored by fluorescence IVFC were not distributed homogeneously in the circulation

**Fig. 1:**
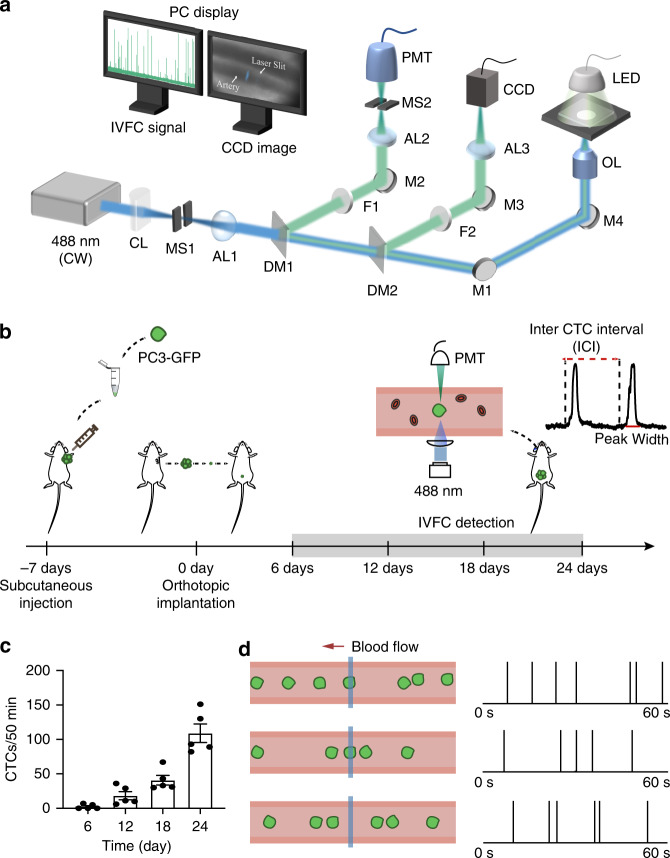
Monitoring prostate cancer CTCs by fluorescence IVFC. **a** Schematic of the fluorescence IVFC experimental setup. CL: cylindrical lens; MS1-MS2: mechanical slits; AL1-AL3: achromatic lenses; DM1-DM2: dichroic mirrors; M1-M4: mirrors; F1-F2: bandpass filters; OL: objective lens; CCD: charge-coupled device; PMT: photomultiplier tube. **b** Schematic of the workflow to establish an orthotopic mouse model of prostate cancer and perform IVFC detection. A 488-nm laser is focused across the ear of a mouse with an objective lens. The fluorescence emission of a GFP-labeled CTC is collected by a PMT and converted to an electrical signal as a spike above the baseline. **c** Dynamic changes in CTC count during cancer progression. The data are presented as the mean SEM, *n* = 5. **d** Representative examples show that CTCs are not homogeneously distributed in the circulation. Right: IVFC signals detected over 60 s. Each vertical line represents a CTC signal

To explore the temporal distribution of CTCs in the circulation, we established a fluorescence in vivo flow cytometer (Fig. 1a) and an orthotopic nude mouse model of human prostate cancer (Fig. 1b). Fluorescence IVFC was performed on the ear of each mouse. When a GFP-expressing CTC passed through the focused laser sheet across an artery, its fluorescence was excited and collected by a photomultiplier tube (PMT)^22,26^. Thus, the time point of each CTC was recorded. Among five mice bearing prostate tumors, two mice had CTCs on day 6 after tumor implantation. The average CTC counts within 50 min were 2.4 ± 1.5, 18.4 ± 5.9, 75.4 ± 21.0, and 102.0 ± 18.5 on day 6, day 12, day 18, and day 24 after tumor implantation, respectively (Fig. 1c). When analyzing the graphed IVFC data, we observed that the CTC count fluctuated on a short timescale (Fig. 1d), which prompted us to further investigate the temporal dynamics of CTCs.

### The probabilities of bursting activity were higher at the early stages

**Fig. 2:**
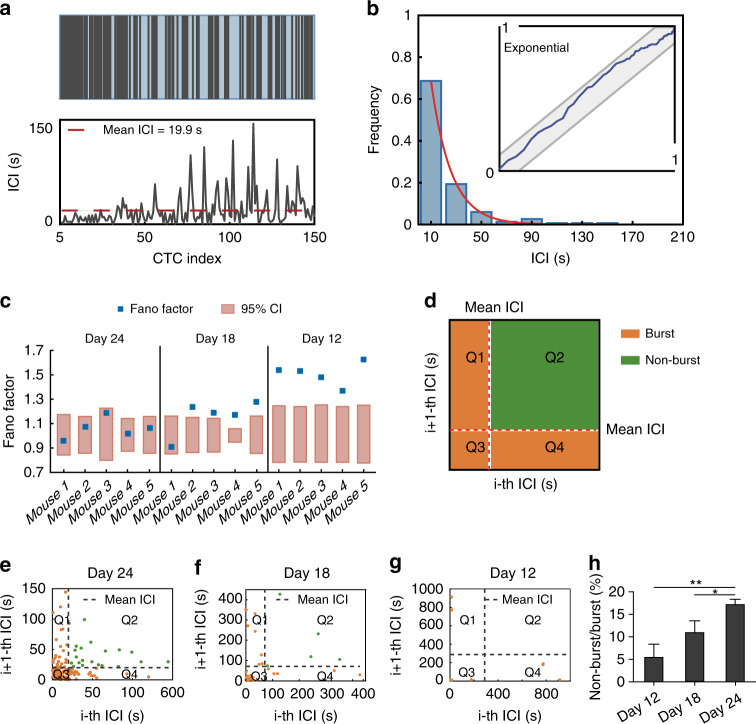
Analysis of the pattern of CTC occurrence at short timescales by fluorescence IVFC. **a** Upper, 151 CTCs detected in 3000 s in a mouse at 24 days after tumor implantation. Lower, ICI of each CTC. The red dashed line shows the average ICI. CTC index is the CTC serial number. **b** ICI histogram with time bins size = 20 s. The inset shows the KS plot of ICIs stays within the 95% confidence band (CI) (gray band), indicating the ICIs follow an exponential distribution. **c** The Fano factor of each mouse on day 24, day 18, and day 12 after tumor implantation. The orange boxes indicate the 95% confidence intervals of the Fano factors in a Poisson process. The small blue boxes indicate the Fano factor of each mouse. **d** Diagram of the joint ICI plot. **e–g** Joint ICI scatter plot on day 24 (**e**), day 18 (**f**), and day 12 (**g**). The *x-*axis shows the current ICI, and the *y-*axis shows the next ICI. The dashed line indicates the mean ICI. **h** The ratio of nonbursts to bursts on day 12, day 18, and day 24; n = 5 mice, unpaired *t*-test. *P < 0.05, **P < 0.01. All data are presented as the mean SEM

To analyze the dynamics of CTCs at short timescales, we first calculated the inter-CTC intervals (ICIs), representing the time intervals between two neighboring CTCs. As shown in Fig. 2a, a total of 150 CTCs were detected within 3000 s in one mouse on day 24, the average ICI was 19.9 s, whereas the ICIs varied from the shortest (less than 1 s) to the longest (more than 144 s). The ICIs of each mouse were visualized by a histogram in which more than 65% of CTC spikes were separated by time intervals of 10 s (Fig. 2b). The distribution of the ICIs was compared with an exponential distribution using the Kolmogorov-Smirnov (KS) test. The ICI probability densities on day 24 lay in the 95% confidence interval of the KS plot, suggesting that the ICIs had an exponential distribution (Figs. 2b and S1). However, on day 18 and day 12, the ICI distribution deviated from an exponential distribution (Figs. S2, S3). This was surprising because CTC occurrence was previously inferred as a Poisson process^27,28^.

To rigorously test whether the occurrence of CTCs was a Poisson process, the Fano factor (FF) was computed as the ratio between the variance in the CTC count and the mean^29^. The Fano factor for a Poisson process falls within the 95% confidence interval around the value of 1^30^. The Fano factors of the 5 mice on day 24 fell within the 95% confidence interval (Fig. 2c), suggesting that CTC occurrence was a Poisson process. However, on day 18 and day 12, the Fano factors were well above the 95% confidence interval, except for mouse 1 on day 18 (Fig. 2c), indicating greater variation in CTC occurrence than could be accounted for in a Poisson process at early stages.

We also observed stochastic bursts of CTCs that occurred near each other and interspersed with periods that contain fewer CTCs (Figs. S1–S3, upper panels). To measure the bursting activity at different stages of cancer, we compared the nonburst-to-burst ratio on day 12, day 18 and day 24. The bursts were defined as two or more successive CTCs whose ICIs were smaller than the mean ICI^31^. We constructed a joint ICI plot, which plotted the current ICI (i-th ICI) against the next ICI (i + 1-th ICI) (Figs. 2e–g, S1–S3). We found that the probabilities of the bursting activity were higher on day 12 and day 18 than on day 24(Fig. 2h). Taken together, these results showed that CTC occurrence at early stages was more variable than expected for a Poisson process. The bursting behavior observed in CTCs might explain the non-Poisson variations.

### CTC counts exhibited remarkable daily oscillations revealed by fluorescence IVFC

**Fig. 3:**
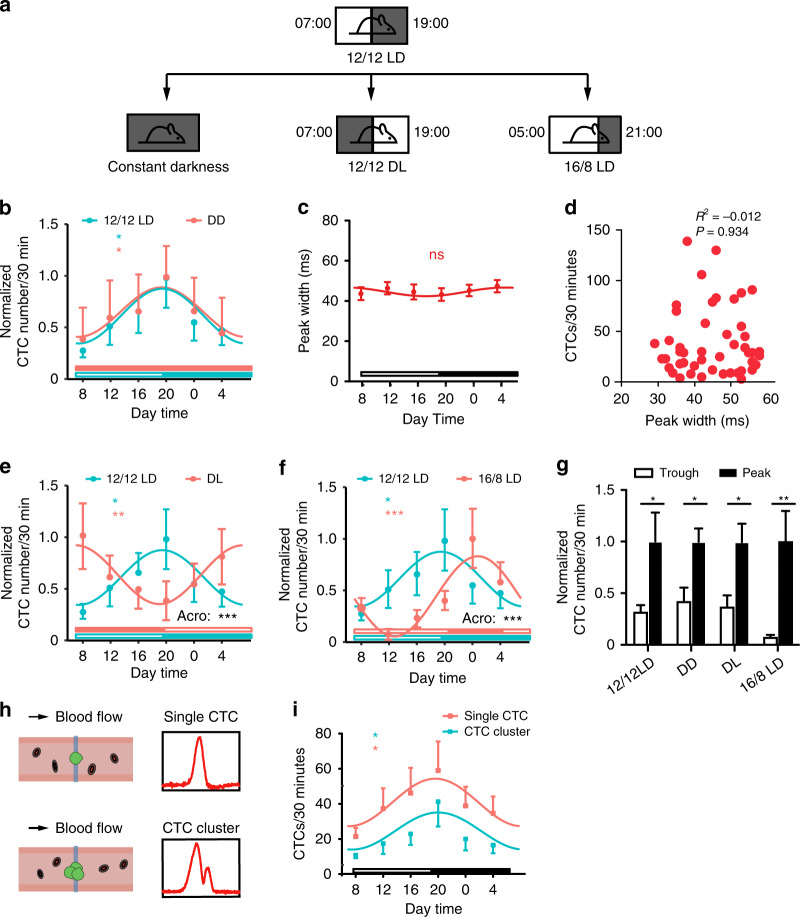
CTC counts exhibited circadian oscillation in mice monitored by fluorescence IVFC. **a** Schematic of the light-dark cycle. **b** Circadian rhythm of CTCs in mice maintained under 12/12 LD and DD conditions. Unfilled bars: light phase, filled bars: dark phase. The statistical significance of the fluctuation was evaluated using Cosinor analysis, *n* = 5–8 mice. **c** Daily variations in the peak widths of the IVFC signals detected in mice maintained under 12/12 LD conditions. Cosinor analysis revealed no significant fluctuation, *n* = 8 mice. **d** Pearson correlation analysis of the CTC count with the peak width (*R*^2^ = −0.012, *P* = 0.934). **e** Circadian rhythm of CTCs in mice maintained under 12/12 LD and DL conditions. The statistical significance of the fluctuation was evaluated using Cosinor analysis, *n* = 3–8 mice. There was a significant difference in acrophase (Acro) between the 12/12 LD and DL groups. **f** Circadian rhythm of CTCs in mice maintained under 12/12 LD and 16/8 LD conditions. The statistical significance of the fluctuation was evaluated using Cosinor analysis, *n* = 6–8 mice. There was a significant difference in acrophase (Acro) between the 12/12 LD and 16/8 LD groups. **g** CTC counts at the peak and trough of the rhythm, *n* = 3–8 mice, unpaired *t*-test. **h** Schematic diagram showing the fluorescence IVFC signals of a single CTC and a CTC cluster. **i** Daily variation in the numbers of single CTC and CTC clusters detected in mice maintained under 12/12 LD conditions. *P < 0.05, **P < 0.01, ***P < 0.001. All data are presented as the mean SEM

To elucidate the daily variation in CTCs, mice were housed under a 12 h light:12 h dark cycle (12/12 LD) with lights-on at 07:00 and lights-off at 19:00 (Fig. 3a). The lights-on time was defined as zeitgeber time 0 (ZT0). Fluorescence IVFC was performed to monitor CTCs at 4-h intervals starting at 08:00 (ZT1) to 04:00 (ZT21). Circadian rhythmicity was determined using Cosinor analysis with MetaCycle^32^. We found that the numbers of CTCs significantly oscillated in peripheral blood, exhibiting a peak at 20:00 (ZT13) and a trough at 08:00 (ZT1) (Fig. 3b). Since blood flow velocity could affect the number of CTCs, we assessed whether the blood flow velocity varied during a 24-h period. The peak width of the fluorescence IVFC signal is determined by cell velocity. We calculated the peak width of each CTC at different time points and found no significant daily variation (Fig. 3c). Pearson correlation analysis revealed no significant correlation between the peak width and the CTC count, suggesting that the daily variation in CTCs was not caused by a change in the blood flow velocity (Fig. 3d). To reveal the endogenous rhythm of CTCs, mice were transferred to constant darkness (DD) for two weeks to eliminate the entrainment effects of light on the circadian clock^33^ (Fig. 3a). The rhythmic oscillations of CTCs were sustained in DD, indicating the bona fide endogenous nature of CTCs (Fig. 3b)^34,35^.

Since light is a major entrainment cue of the circadian clock, we next questioned whether light was an entraining signal for the daily oscillations of CTCs. We induced changes in the light regime by switching mice to an inverted light cycle (DL) or a 16 h light:8 h dark cycle (16/8 LD) for a minimum of 2 weeks to completely establish the changing light cycle before detection as previously reported^34,36^ (Fig. 3a). The differences in amplitude, midline-estimating statistic of rhythm (MESOR), and acrophase of the rhythm between the two groups were compared^37^. Notably, the DL cycle fully inverted the acrophase of CTC oscillations (Fig. 3e). Furthermore, the 16/8 cycle induced a 4-hour shift in the acrophase (Fig. 3f). Together, these data indicated that the light-dark cycle was an entraining cue of the CTC rhythm, which could fully reset and entrain their intrinsic circadian rhythm. The significant daily fluctuation of the CTC counts led to a 2-fold to 12-fold change in numbers between the peak and trough of the CTC rhythm in the four light and dark conditions (Fig. 3g). In addition, we found no significant difference between the rhythm of CTC clusters and single CTCs when we analyzed the fluorescence signal pattern (Fig. 3h, i, Movies S1, S2).

### Larger blood volume and multiple small samples improved CTC detection accuracy

**Fig. 4:**
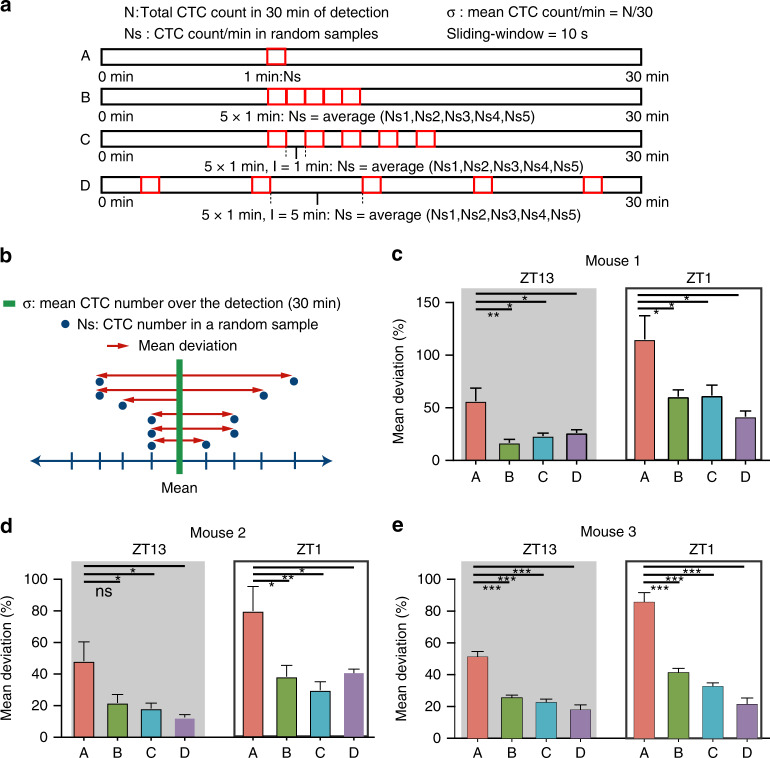
Multiple sampling improves detection accuracy. **a** Schematic of different sampling strategies. **b** Diagram of computing the mean deviation. **c–e** Mean deviation of different sampling strategies, unpaired *t*-test. *P < 0.05, **P < 0.01, ***P < 0.001. All data are presented as the mean SEM

Considering that the numbers of CTCs varied at short and long-time scales, we aimed to optimize the sampling strategy to increase the detection accuracy of CTCs by the in vivo data. We questioned whether the diagnostic yield of CTC detection was correlated with the sampling volume and sampling time. We evaluated the detection accuracy of a random sample of four sampling strategies at ZT13 and ZT1 (Fig. 4a). The A strategy assessed 1 min of sample; the B strategy assessed 1 min of sample 5 times, with no time interval between each sample, and averaged the values; the C strategy assessed 1 min of sample 5 times, with 1 min between each sample, and averaged the values; the D strategy assessed 1 min of sample 5 times, with 5 min between each sample, and averaged the values.

To quantify the detection accuracy, we computed the deviation of CTC count obtained in a random sample from the mean CTC count calculated from the 30 min data (hereafter, the “mean deviation”) (Fig. 4b). Therefore, the smaller the mean deviation was, the higher the detection accuracy of CTCs. The mean deviation was lower at ZT13 than at ZT1, suggesting that the sampling time influenced the detection accuracy. In addition, multisampling decreased the mean deviation (Fig. 4c–e). These results indicated that multisampling and sampling at ZT13 would improve the accuracy of CTC detection. As the B strategy was equivalent to taking a 5 min sample, a larger blood volume would also improve the detection accuracy.

## Discussion

Even though great efforts have been made, the mechanisms underlying cancer metastasis remain largely unknown. The CTC count and its biological rhythm have important implications for cancer treatment and prognosis. However, the detection of CTCs in the blood is unpredictable. In this study, we performed fluorescence IVFC to investigate the temporal distribution of CTCs. We found stochastic bursts and a strong circadian rhythm of CTC release. The CTC count in a certain volume was considered to follow a Poisson distribution^27,38^. We found that CTCs occurred as a Poisson process on day 24 after tumor implantation. However, when CTCs were rare at earlier stages, they tended to occur in bursts. These clusters of cells might travel together to increase survival and co-home to form polyclonal metastases^39^. We speculate that the large number of CTCs at the late stages of the disease conceals the burst activity. Nonetheless, CTC count is more variable than expected for a Poisson process.

Compared with the current in vitro detection methods of CTCs, which involve drawing blood and are based on biophysical and biochemical principles, in vivo optical techniques have shown great advantages according to their higher temporal resolution and noninvasive characteristics. These advantages of optical detection enable the monitoring CTCs at a series of time points during the day/night cycle. As a widely used but still developing optical technique, this work extends an important application range of fluorescence IVFC in the study of the circadian rhythm of CTCs. IVFC might be the most suitable optical detection method for assessing CTCs according to research on circulation dynamics in animal models. There are several varieties of IVFC, such as photoacoustic IVFC^40,41^ and Raman IVFC^42–44^. They have the capability to detect label-free CTCs in vivo, which is based on the contrast of endogenous biomarker of CTCs, such as melanin in circulating melanoma cells. Although these label-free IVFC techniques are limited to only a few types of cancer due to the low concentration of cancer biomarkers in CTCs, it is foreseeable that there will be breakthroughs in the near future with their rapid optical development in biomedical research.

Studies in humans have disclosed conflicting conclusions regarding circadian variations in CTCs. A study focused on multiple myeloma observed robust circadian fluctuation of CTCs^45^, whereas two studies on patients with metastatic breast cancer found no significant differences in CTC counts between daytime and nighttime^46,47^, but 87% of patients in these studies received periodic steroid treatment, which may disrupt the circadian rhythm and thus influence the cell cycle and dissemination^48,49^. Moreover, the absence of daily variation in CTC counts in those studies may, at least partially, be due to the low sensitivity of the isolation technologies. In addition, blood samples were collected twice a day at 08:00 and 20:00 in their studies, and this interval might be too long to characterize the daily oscillations in CTCs. Given that CTCs were rare and their counts could fluctuate periodically during the day, as shown in this study, there might be no significant difference between CTC counts at two timepoints far from each other. Considering that the daily oscillation and short-term fluctuation of CTCs are likely to increase the uncertainty of in vitro detection, collecting samples at different time points over 24 h and assessing larger blood volumes or multiple small samples could help to improve detection accuracy.

There are two potential explanations for why CTC counts are regulated by the circadian rhythm: physical factors and biological factors. Mice are nocturnal animals that are active during the night phase and rest during the light phase^50–53^, both in physical activities and biological processes. A study showed that CTC dynamics changed after physical pressure and palpation^54^. Mice are more likely to be affected by physical pressure on the tumor during the active night phase. Although there are quite a few studies investigating the relationship between CTC counts and animal behavioral/physical activities, it is worth conducting more in-depth studies on this topic. Compared to physical factors, biological factors seem to play more important roles in regulating CTC release during the dark and light phases. For example, the immune functions vary over a day^55^. Most mature immune cells, including natural killer (NK) cells, are released into the blood at the beginning of the rest phase and migrate to organs during the active phase^56^. As part of the innate immune system, NK cells play a central role in the intravascular anti-tumor response^57^. During the rest phase, the number of NK cells in the blood increases^58^, which may result in a low level of CTCs. The number of NK cells reaches the lowest level at the onset of the active phase, which may lead to the peak of CTC counts.

Epithelial-mesenchymal transition (EMT) has been implicated to be responsible for the release of tumor cells from the primary tumor^59^. The successful shedding of tumor cells into the vessels involves interplay between the tumor and microenvironment. As an important part of the tumor microenvironment, macrophages secrete tumor necrosis factor-α (TNF-α) to promote the EMT of cancer cells^60^. The amount of TNF-α in macrophages peaks around the onset of the active phase^61^, suggesting that more CTCs can be released at the active phase. Therefore, the shedding of tumor cells may be related to the active and rest cycle.

Our data demonstrate that the number of CTCs peaks at the onset of the active phase, which indicates that the trafficking of CTCs is regulated by the circadian clock. Further systematic investigations are needed to address whether the circadian clock influences the shedding process, survival, and/or extravasation process of CTCs. Our results suggest that rest (sleep) is important and that a lack of sleep may increase CTC release and enhance metastasis risk. As the tolerability and efficacy of anticancer therapies are related to the circadian rhythm^62^, the CTC rhythm may help optimize treatment timing and improve the results of chronotherapy.

Overall, this work reveals time-of-day differences and short-term variations in CTC trafficking. Our findings suggest that hematogenous metastasis may be regulated by circadian rhythm-related genes and that developing time-of-day specific treatment may help to improve treatment outcomes.

## Materials and methods

### Cells and animal models

The human prostate cancer cell line PC3 was a gift from Prof. Wei-Qiang Gao (School of Biomedical Engineering and Med-X Research Institute, Shanghai Jiao Tong University). Cells were cultured in RPMI-1640 medium with 10% fetal bovine serum (Gibco, Waltham, MA, USA). PC3 cells were transfected with pLVX-EGFP1-C1 (Takara, Mountain View, CA). Polybrene (Takara, Mountain View, CA) was added according to the manufacturer’s instructions.

All mice were housed under 12/12 LD conditions with food and water ad libitum for two weeks before tumor implantation. BALB/c nude mice (male, 4–6 weeks) were used in this study. A total of 1 × 10^6^ PC3-GFP cells suspended in 100 μL PBS were injected subcutaneously into the left flank of recipient mice. The formed subcutaneous tumors were cut into small cubes (~1 mm^3^). The mice were anesthetized with isoflurane. The operation area was sterilized with iodophors and 75% medical-grade alcohol. A lower midline abdominal incision was made, and the bladder and seminal vesicle were externalized using a dry sterilized cotton swab. The dorsal prostatic lobe was exposed by tilting back the seminal vesicles. The prostate capsule was opened with an ~1 mm incision, and a tumor cube was inserted into the capsule. Then, the capsule was closed with an absorbable 7–0 suture to prevent the tumor cube from falling out. After that, the muscle layer and skin were closed with 5–0 surgical sutures. All surgical procedures were performed in a sterile environment.

All animal experiments were performed with the approval of the Ethical Committee of Animal Experiments of Med-X Research Institute and School of Biomedical Engineering at Shanghai Jiao Tong University.

### Fluorescence IVFC

An experimental in vivo flow cytometer was established in our laboratory, as illustrated in Fig. 1a. Specifically, the light source was a 488-nm laser, which was reshaped into a light sheet using a cylindrical lens. The light sheet was focused by an objective lens and positioned across a mouse artery. The artery was illuminated with a 532-nm LED and imaged with a charge-coupled device (CCD). When a GFP-labeled CTC passed through the laser slit, its fluorescence was excited and collected by a PMT. The mouse was anesthetized with 100 mg ketamine and 10 mg/kg xylazine and placed on the sample stage with its ear adhered to a microscope slide. Fluorescence IVFC was performed on each mouse (*n* = 5) for 50 min every 6 days from day 6 to day 24. Data on day 18 and day 24 were used for ICI analyses. For 24-h monitoring, fluorescence IVFC was performed on each mouse (*n* = 8) for 30 min every 4 h from ZT1 to ZT21 at day 18.

We also discriminated CTC clusters with single CTCs through the signal pattern detected by fluorescence IVFC. Signals with multiple peaks were produced by CTC clusters, while single-peak signals were produced by single CTCs^26^ (Fig. 3f).

### Fluorescence IVFC data analyses

Raw data acquired by fluorescence IVFC were analyzed with MATLAB. First, the data were denoised and identified according to our previous work^26^. Then, the peak height and width were calculated and stored.

### Poisson process test

A Poisson process is defined as a counting process in which the inter-events-intervals have an exponential distribution. To test whether CTC occurrence was a Poisson process, we first determined whether the ICIs were exponentially distributed. The empirical cumulative density function (CDF) was compared with a model CDF of the exponential based on the KS test^63^. The CDF of the exponential distribution was

1 $$F\left( {x;\lambda } \right) = \left\{ \begin{array}{ll}1 - e^{ - \lambda x}, & x \ge 0\\ 0, & x\, < \,0\end{array} \right.$$

where is the intensity of CTC occurrence, was estimated by the maximum log-likelihood

2 $$L = C \times log \left( \lambda \right) - \lambda \times \mathop {\sum}\limits_{i = 1}^n {{ICI}_i}$$

where *C* is the number of ICIs. The maximum difference between CDFs was asymptotically distributed and was used to calculate the 95% CIs. A well-fit model should stay entirely within these bounds. Then, the Fano factor was calculated as the sample variance divided by the sample mean to compare the variability from CTC occurrence with a Poisson process. The 95% CI of the Fano factor was calculated as previously reported^30^. The Fano factor for a Poisson process should lie within the 95% CI.

### Burst identification

The mean ICI for each mouse at different time points was computed. CTC burst activity was defined as two or more successive CTCs whose ICIs were smaller than the mean ICI^31^. To construct the joint ICI plot, the mean ICI for each mouse was computed, and the *xy*-plane was divided into four sections: the dots in Q1 represented final CTCs in bursts; the dots in Q2 represented sporadic CTCs; the dots in Q3 represented CTCs within bursts; and the dots in Q4 represented initial CTCs in bursts. Therefore, the dots in Q1, Q3, and Q4 were bursting CTCs, while the dots in Q2 were nonbursting CTCs.

### Detection accuracy measurement

The mean deviation was computed as

3 $${{Mean}}\,{{deviation}} = \frac{{\left| {Ns - \sigma } \right|}}{\sigma } \times 100\%$$

where σ represents the mean count of CTCs in 30 min and Ns is the CTC count in 1 min or 5 min of detection.

The coefficient of variation (CV) was computed as

4 $${{CV}} = \frac{Std}{{mean}}$$

### Statistical analyses

Statistical analyses were performed with GraphPad Prism. Data were presented as the mean SEM. Circadian rhythmicity significance was evaluated by Cosinor analysis using Metacycle^32^, an R package. The differences in amplitude, MESOR, and acrophase between the two groups were compared using the Cosinor2 package^37^.

## Supplementary information

Movie S1

Movie S1

Supplementary information
