# Supplementary material for: In vivo flow cytometry reveals a circadian rhythm of circulating tumor cells
Source: Light Sci Appl. 2021 May 28;10:110. doi: 10.1038/s41377-021-00542-5 (PMC8160330; doi:10.1038/s41377-021-00542-5)
Supplement: Supplementary file 3 — Supplementary information [file 41377_2021_542_MOESM3_ESM.docx]

*Supplementary Information for:*

*In vivo* flow cytometry reveals a circadian rhythm of circulating tumor cells

Xi Zhu^1,#^, Yuanzhen Suo^2,3,#,*^, Yuting Fu^1^, Fuli Zhang^1^, Nan Ding^1^, Kai Pang^4^, Chengying Xie^1^, Xiaofu Weng^1^, Meilu Tian^5^, Hao He^1,*^, Xunbin Wei^1,5,6,*^

^1^ State Key Laboratory of Oncogenes and Related Genes, Shanghai Cancer Institute, Med-X Research Institute and School of Biomedical Engineering, Shanghai Jiao Tong University, Shanghai 200030, China;

^2^ Biomedical Pioneering Innovation Center, Peking University, Beijing 100871, China;

^3^ School of Life Sciences, Peking University, Beijing 100871, China;

^4^ School of Instrument Science and Optoelectronics Engineering, Beijing Information Science & Technology University, Beijing 100192, China;

^5^ Biomedical Engineering Department, Peking University, Beijing, 100081, China;

^6^ Key Laboratory of Carcinogenesis and Translational Research (Ministry of Education/Beijing), Peking University Cancer Hospital & Institute, Beijing, 100142, China;

^#^ These authors contribute equally to this work.

^*^ Corresponding author: Dr. Yuanzhen Suo, Integrated Research Building, 5 Yiheyuan Road, Beijing 100871, China. E-mail: suoyuanzhen@pku.edu.cn, Prof. Hao He, Med-X Research Building, 1954 Huashan Road, Shanghai 200030, China. E-mail: [haohe@sjtu.edu.cn](mailto:haohe@sjtu.edu.cn). Prof. Xunbin Wei, Med-X Research Building, 1954 Huashan Road, Shanghai 200030, China. E-mail: [xwei01@sjtu.edu.cn](mailto:xwei01@sjtu.edu.cn)

**
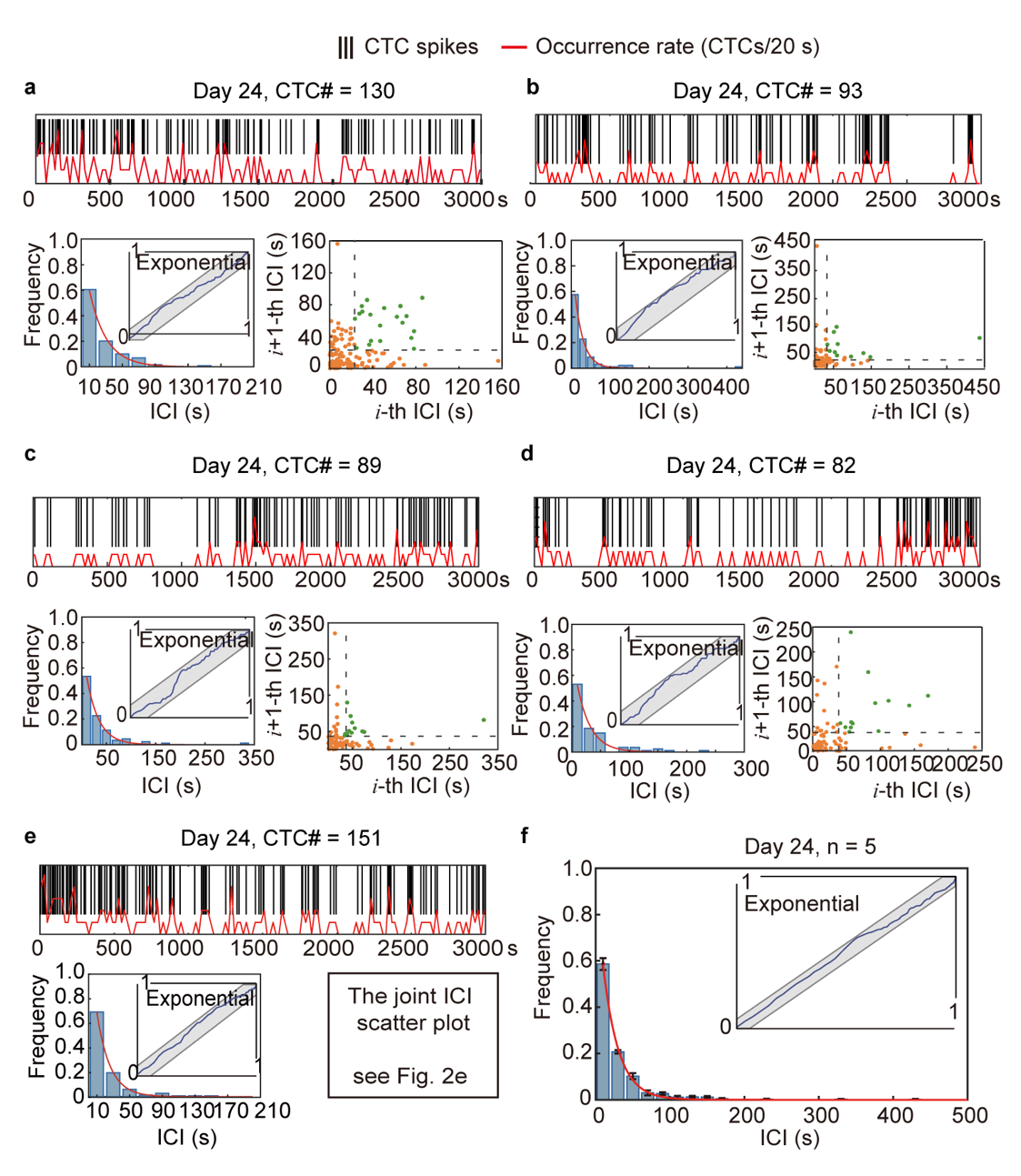
**

**Fig. S1** Analysis of CTC distribution of each mouse on Day 24. **a-e**, Upper, the raster plot and firing rate (red) of the IVFC signals. Lower left, ICI histogram with time bins size = 20 s. The inset shows the KS plot (blue curve) of ICI distribution with exponential distribution and the 95% confidence band (gray band). Lower right, The joint ICI scatter plot. **f**, ICI histogram and KS plot of the pooled data; n = 5 mice.

**
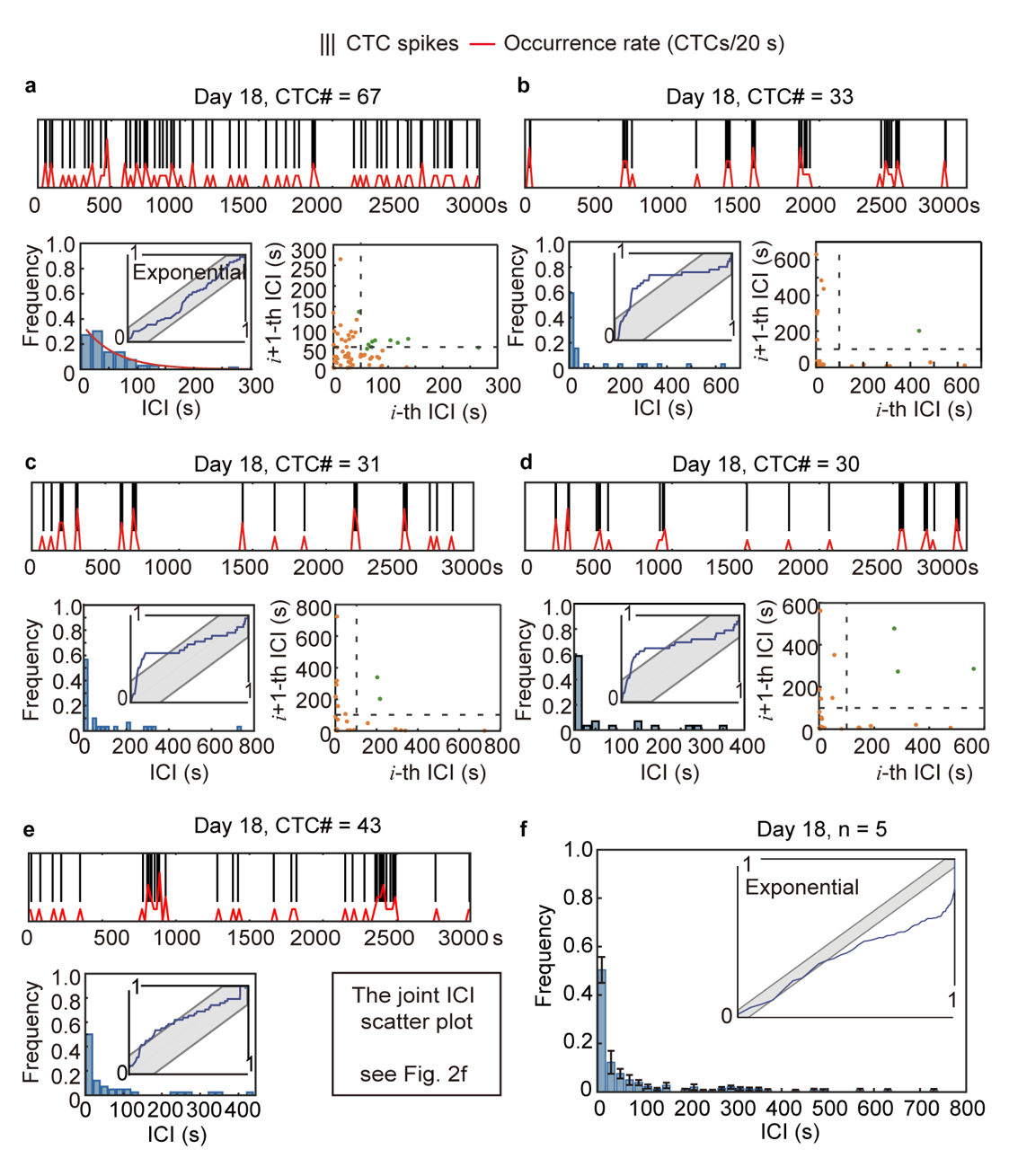
**

**Fig. S2** Analysis of CTC distribution of each mouse on Day 18. Upper, the raster plot and firing rate (red) of the IVFC signals. Lower left, ICI histogram with time bins size = 20 s. The inset shows the KS plot (blue curve) of ICI distribution with exponential distribution and the 95% confidence band (gray band). Lower right, The joint ICI scatter plot. **f**, ICI histogram and KS plot of the pooled data; n = 5 mice.

**
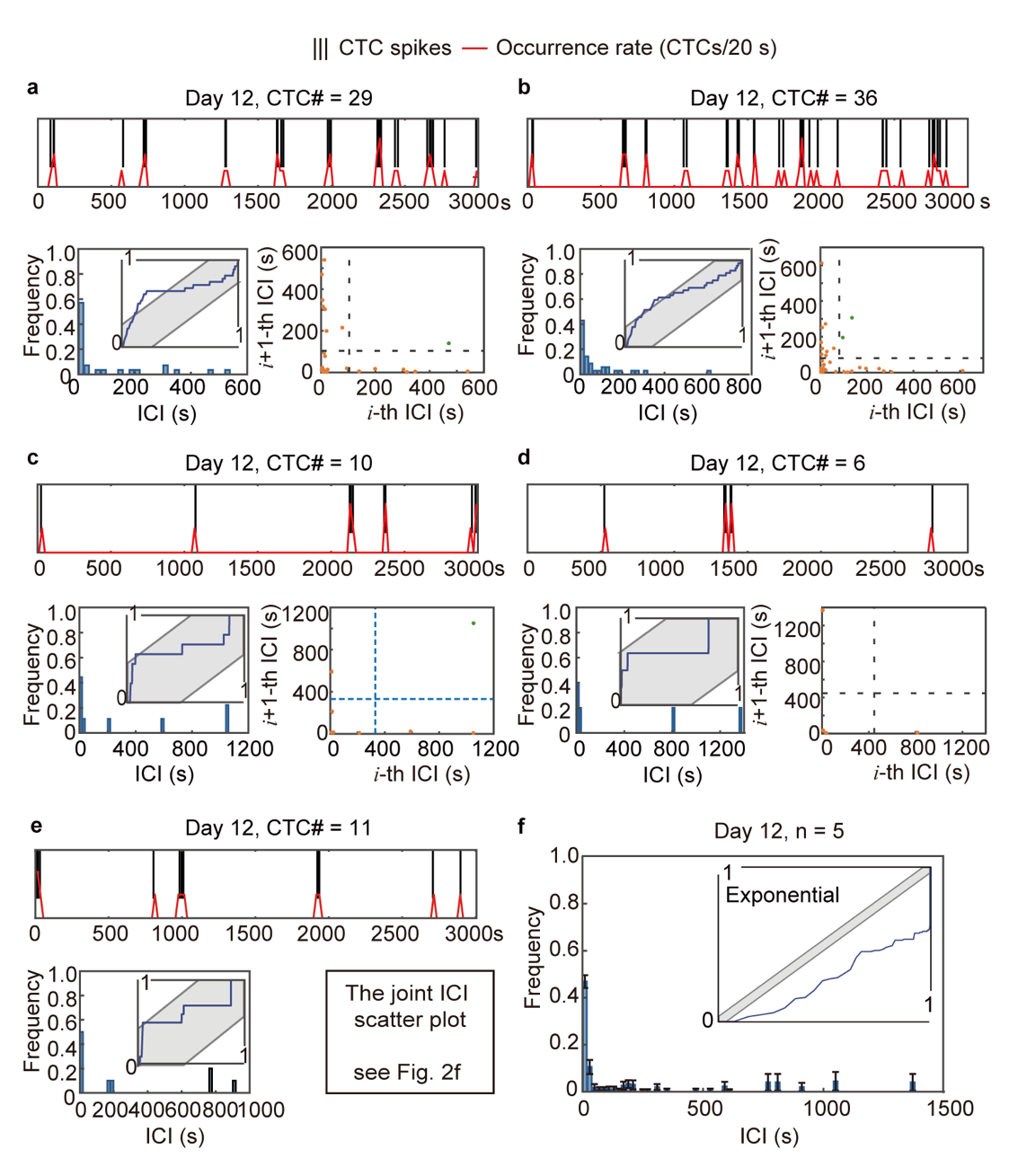
**

**Fig. S3** Analysis of CTC distribution of each mouse on Day 12. Upper, the raster plot and firing rate (red) of the IVFC signals. Lower left, ICI histogram with time bins size = 20 s. The inset shows the KS plot (blue curve) of ICI distribution with exponential distribution and the 95% confidence band (gray band). Lower right, The joint ICI scatter plot. **f**, ICI histogram and KS plot of the pooled data; n = 5 mice.

**Movie S1**

In vitro simulation of IVFC signals produced by a single CTC in Fig. 3i. In the videos, a syringe pump and a glass capillary are used to simulate the blood flow. Cells are pumped by the syringe pump at a flow rate of the mouse ear artery (~ 10 mm/s). The scale bar is displayed in the video.

**Movie S2**

In vitro simulation of IVFC signals produced by a CTC cluster in Fig. 3i. The same simulation method with that in supplementary video 1.
